# Extended Calix[4]arene-Based Receptors for Molecular Recognition and Sensing

**DOI:** 10.3390/s8095313

**Published:** 2008-09-01

**Authors:** Pik Kwan Lo, Man Shing Wong

**Affiliations:** Department of Chemistry and Centre for Advanced Luminescence Materials, Hong Kong Baptist University, Kowloon Tong, Hong Kong SAR, P.R. China

**Keywords:** Extended calix[4]arene, cooperative binding, recognition, sensing

## Abstract

Recent advances in the area of recognition and sensing have shown that artificial receptors derived from extended calix[4]arenes bearing multiple π-conjugated fluorophoric or chromophoric systems have found useful to enhance binding affinity, selectivity and sensitivity for recognition and sensing of a targeted ion or molecule. A comprehensive review of various π-conjugation-extended calix[4]arene-based receptors with the highlight on the design and binding characterization for recognition and sensing is presented.

## 1. Introduction

There is considerable interest in developing efficient artificial receptors for molecular recognition and sensing as they play important functional roles in biological, medical, environmental, and chemical sciences [1-8]. There has been significant progress in exploring artificial receptors for sensing, particularly for metal ion sensing over past two decades [9-17]; however, design and synthesis of artificial receptors that exhibit high binding affinity, selectivity, and sensitivity to a targeted molecule or anion still pose a great challenge to the scientific community. Design of complementary structural units that exhibit recognition properties requires building in relatively strong and directional attractive forces such as hydrogen bonding, metal-ligand and electrostatic interactions. Strong binding interaction often provides promise for high sensitivity. As a result, functional groups such as amides [18-22], ureas [23-24], thioureas [25-32], amidoureas [33-37], crown ethers [38-39], carboxylic acids, azacrown ethers [40-42], ester [43-46], and positively charged groups [47-49] have been widely used to append onto artificial receptors for recognizing neutral molecules and ions via non-covalent interactions. Receptors/sensors possessing multiple ligating groups are often found useful to promote cooperative interactions which can result in enhanced binding affinity [50]. On the other hand, a rigid and preorganized receptor site that is complementary to the target guest is also crucial to achieve high binding affinity and good selectivity.

**Figure 1.**
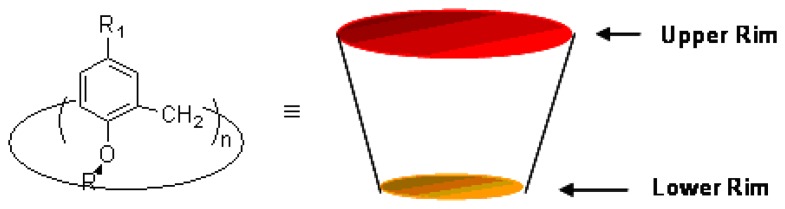
Molecular structure of calix[n]arene

Calix[n]arenes are macrocyclic molecules that are generally derived from the base catalyzed condensation of formaldehyde and phenol [51-56]. Such macrocycles possess a well-defined hydrophobic upper rim and hydrophilic lower rim surrounding a hollow cavity with varied dimensions that depends on the number of the phenolic units incorporated (Figure 1) [57-60].

**Figure 2.**
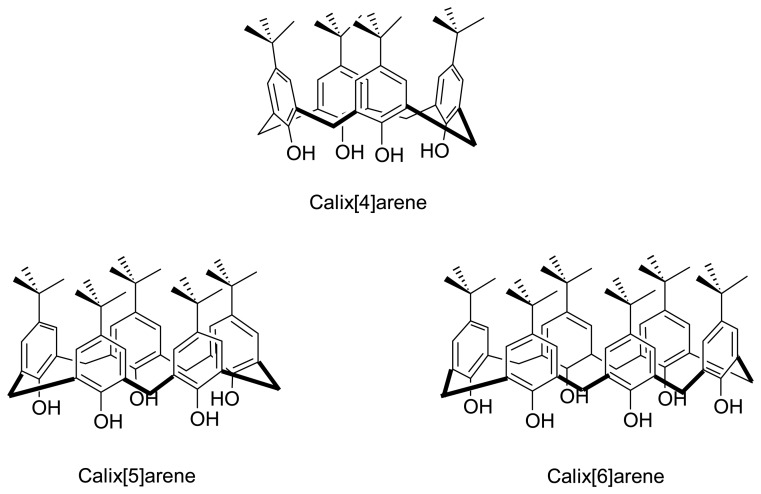
Different sizes of calix[n]arenes

Calix[n]arenes have been explored in depth as building blocks or molecular scaffolds for artificial receptors and supramolecular chemistry over the years because of their unique three-dimensional and tunable structures, together with the ease of functionalization [61-62]. The chemistry and applications of calix[n]arenes have been extensively discussed in several books [51-52, 55] and review articles [63-69]. Much research work has also been reported recently on applications of calix[*n*]arenes in the area of supramolecular chemistry. Calix[n]arenes can act as vase-like building blocks in the construction of artificial receptors for selective recognition of cations, anions, and neutral molecules through principles of molecular recognition. However, large-size calix[n]arenes with n > 4 (Figure 2) possess a high degree of conformational freedom, attempts to apply such calix[n]arenes (n > 4) as hosts for a specific molecular recognition have so far been limited.

**Figure 3.**
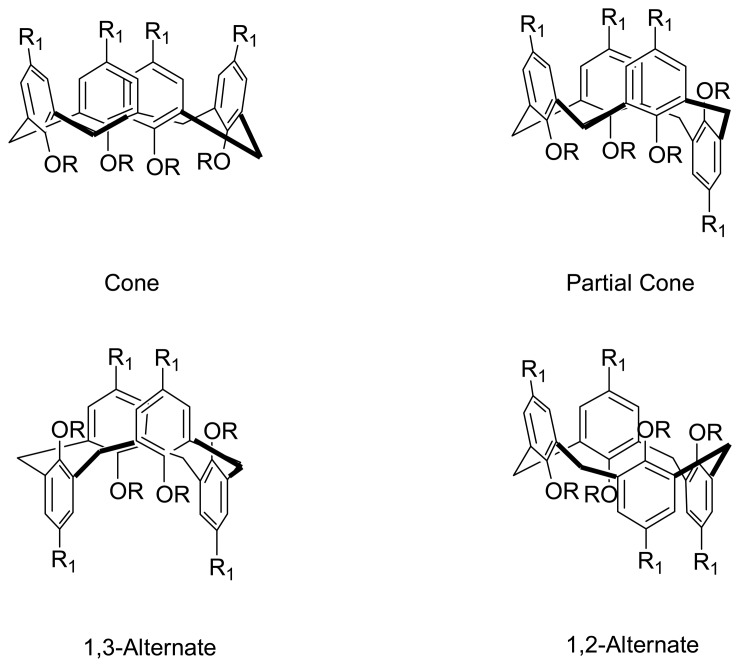
Four different conformations of calix[4]arenes.

As a result, calix[4]arene is of particular interest as a molecular scaffold in the design of artificial receptors because of its stable and unique three-dimensional structure. Calix[4]arene exits in four different conformations namely cone, partial-cone, 1,2-alternate, and 1,3-alternate (Figure 3).

The calix[4]arene platform can be further expanded and deepened by incorporating planar aromatic moieties at the *para*-positions of the phenolic units. Recently, there have been increasingly interests in taking advantage of an extended preorganized rigid platform of calix[4]arene for construction of supramolecular systems [70]. Receptors with the expanded or extended cavity are beneficial to the recognition and encapsulation with which a larger targeted guest can be accommodated. The extended π-conjugated calix[4]arene skeleton can also act as a chromophore or fluorophore for signal display. Upon binding with a guest, the change of the spectral properties of the π-conjugated skeletons would give rise to a sensing mechanism. Furthermore, the multiple ligating groups incorporated onto the proximate multi-π-conjugated calix[4]arene assembly can act cooperatively resulting in an enhanced binding [71-74, 83].

In this review, the focus is essentially upon the design and development of expanded or extended calix[4]arene-based receptors appended with multiple π-conjugated fluorophoric or chromophoric systems at the upper rim for molecular recognition and sensing. This review also restricts the discussion on the two conformational isomers, namely cone, and 1,3-alternate of π-conjugation-extended calix[4]arenes.

## 2. Molecular Recognition Based upon Extended Calix[4]arenes

### 2.1. Extended Calix[4]arene-Based Receptors for Cations

Calix[4]arenes have been particularly attractive for their unique binding characteristics towards alkali metal, alkaline earth, and transition metal ions. The first report on calixarene-based sensor appeared in 1986. Diamond *et al.* [73] demonstrated that **1a** and **1b** were excellent sensors for sodium ion. These sensors showed a potential application in determination of sodium contents in blood. However, these receptors limited to the binding/sensing of small size of ions such as sodium ion. To make use of calix[4]arene as a versatile molecular scaffold for recognition, much progress has been made toward tuning the binding capability and affinity of different metal ions by means of chemical structural modifications over the years. One of the promising approaches is to construct a preorganized and preoriented multi-π-conjugated calix[4]arene molecular assembly bearing multiple ligating units to enhance selective binding.


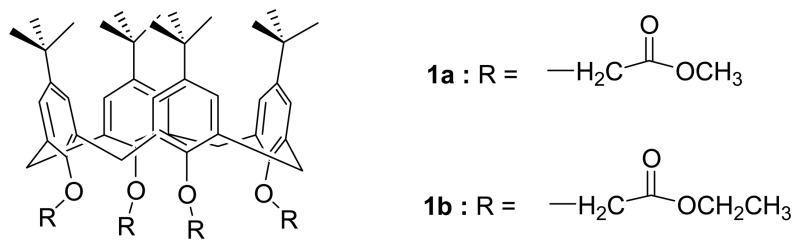


Wong *et al.* [74] have reported a facile and efficient protocol for the synthesis of OPP(n)-substituted calix[4]arene assemblies **2a-c** with n up to 4 via tetraiodophenyl- or tetraiodobiphenyl-calix[4]arenes in which hexylsulfanyl end-substituted OPP(n)-substituted calix[4]arenes exhibit enhanced binding properties for soft metal ions such as Ag^+^ and Hg^2+^.


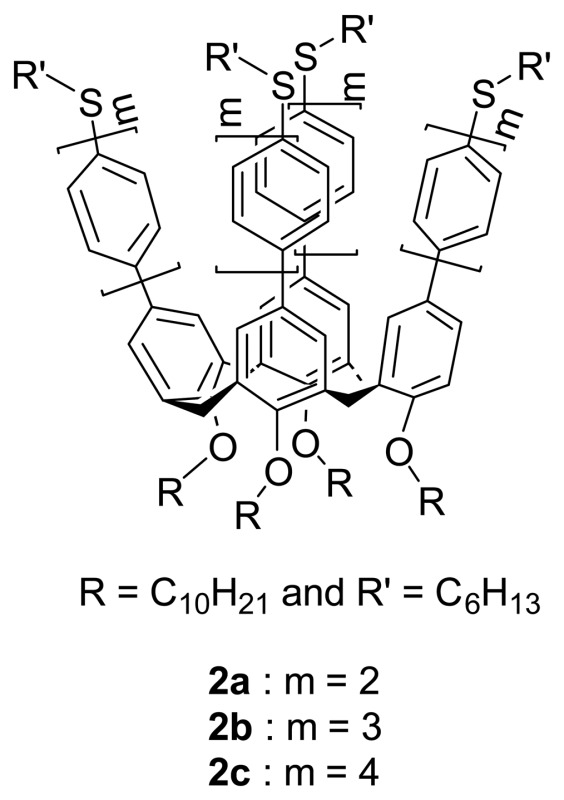


**Figure 4.**
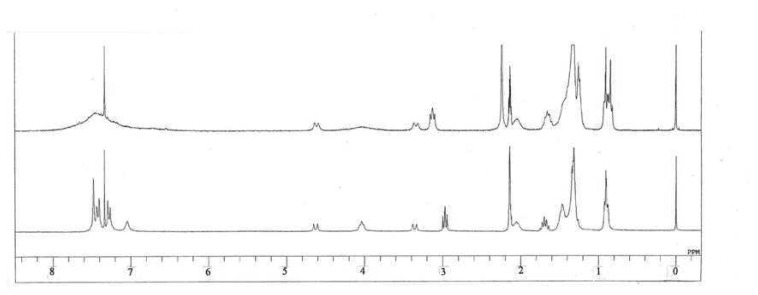
^1^H-NMR spectra of **2b** in CDCl_3_ (bottom) and **2b** with an addition of CF_3_COOAg (top).

Upon addition of Ag^+^ salt in a CDCl_3_/CD_3_COCD_3_ (v/v = 10:1) solution of hexylsulfanyl end-capped OPP-substituted calix[4]arene assemblies, there are dramatic shifts of the proton resonances (Δδ = 0.17-0.25 ppm) and peak broadening/splitting of the methylene protons adjacent to the sulfur atom in the ^1^H-NMR spectra (Figure 4).

**Figure 5.**
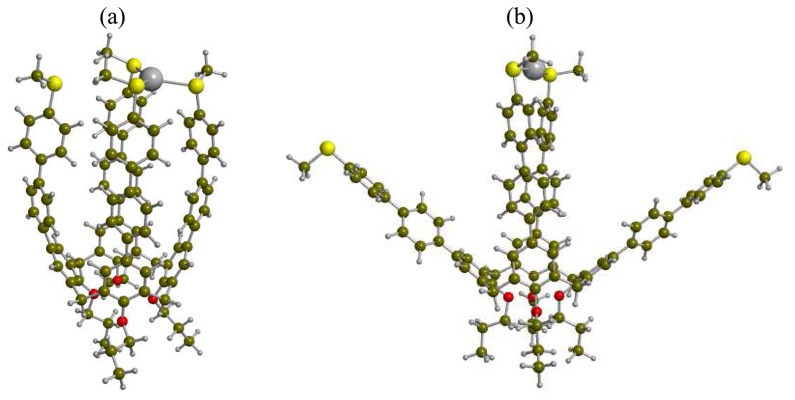
DFT-optimized geometries of an analog of 2·Ag^+^ complex for the (a) pseudo-C*_4v_* binding mode, and (b) near C*_2v_* binding mode. The drawings were performed by MOLDRAW.

The binding stoichiometry of **2a**·Ag^+^ determined by the Job's plot analysis based on the fluorescence titrations and the high-resolution MALDI-TOF MS analyses consistently support a 1:1 binding mode. The binding associations of **2a** and **2b** toward Ag^+^ ion are one to two orders of magnitudes stronger than those of the corresponding monomeric units confirming the importance of the cooperative binding. Figure 5 shows the more stable pseudo-C*_4v_* and less stable near the C*_2v_* binding mode of DFT-optimized geometries of an analogous of **2a**·Ag^+^ complexes.

Wong *et al.* [75] have also demonstrated the use of polyether bridge at the lower rim linked between two distal phenolic rings to suppress the mobility of the cone conformer of oligophenylene-substituted 25,27-dipropoxycalix[4]crown-4s, **3a-d** in order to open up the flattened cavity induced by metal complexation.


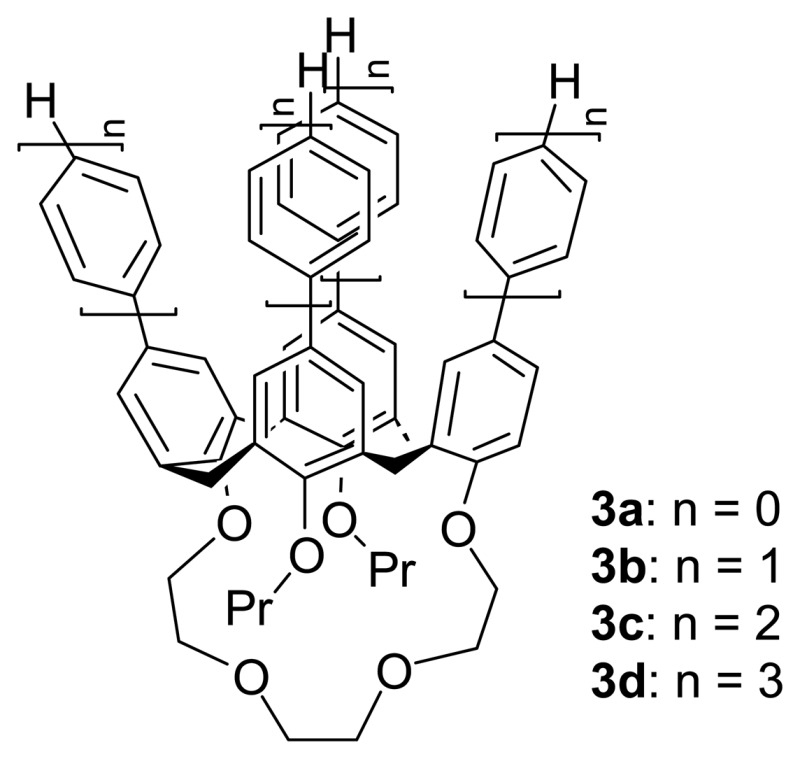


**Figure 6.**
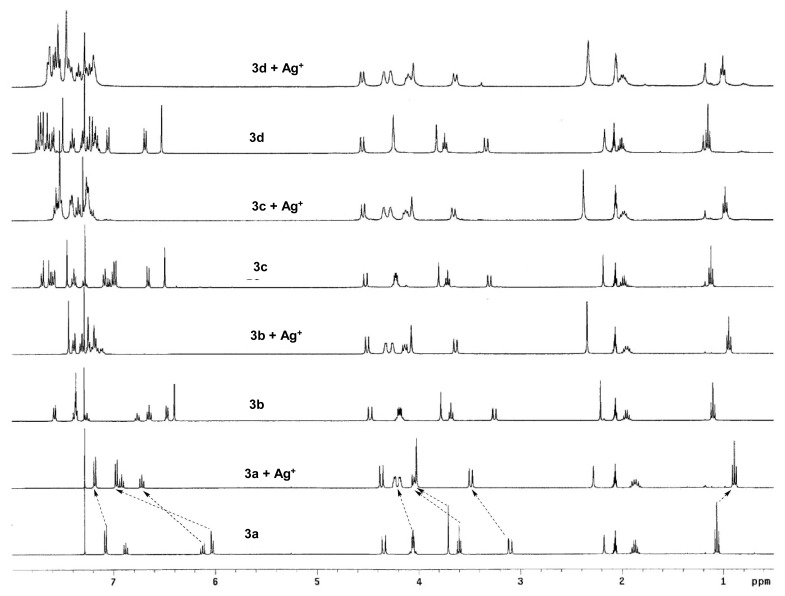
**^1^**H-NMR spectra of calix[4]crown-4s, 3a-d and the corresponding spectra upon addition of 10 equivalents of AgCF_3_COO in CDCl_3_ and CD_3_COCD_3_ (10:1).

The binding interaction of Ag^+^ ion and calix[4]crown-4s was first probed by ^1^H NMR titrations (Figure 6) which was further confirmed with high-resolution ESI-MS or MALDI-TOF MS measurements.

They also investigated the influence of the upper rim functionality on the binding mode and site with a series of *para*-substituted phenyl-calix[4]crown-4s, bearing either methyl or fluoro or trimethylsilyl substituent **4-6**.


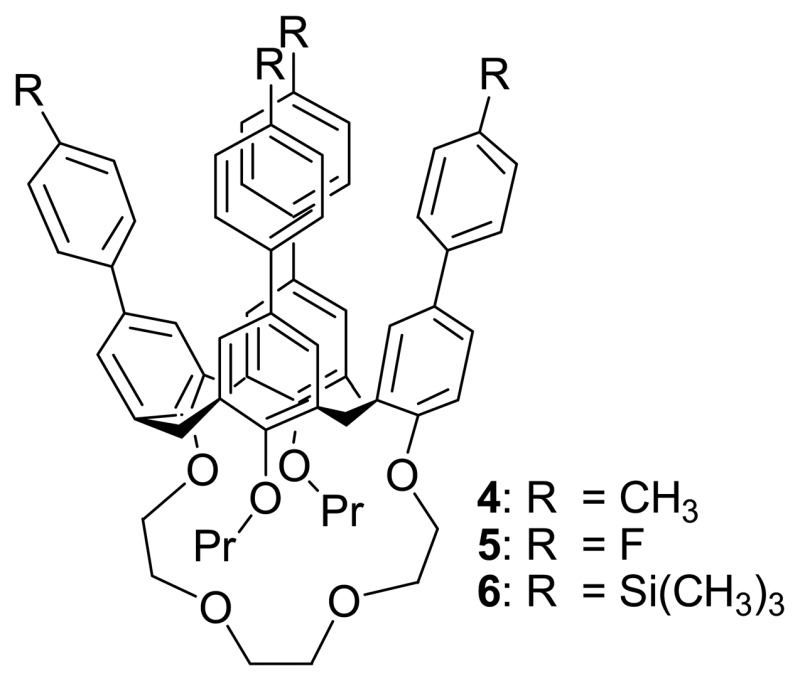


**Figure 7.**
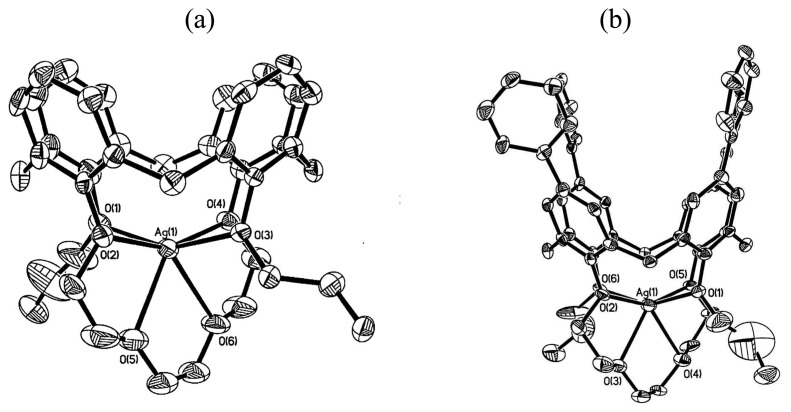
ORTEP drawings of (a) 3a·Ag^+^ and (b) 3b·Ag^+^ (25% probability). All hydrogen atoms are omitted for clarity.

The ^1^H-NMR titration studies showed that the binding was not affected by the electronic and steric properties of aryl rings substituted at the upper rim of calix[4]crown-4s. The single-crystal X-ray structure analyses further reveal that Ag^+^ ion is bound to the ether ligands at the lower rim of calix[4]crown-4s (Figure 7). Because of the weak binding affinity of these calix[4]crown-4s **3a**-**d** toward Ag^+^ ion (K_a_ = 30-91 M^-1^), the dis-assembling process can easily be achieved by stirring the complex with KI at ambient temperature. The complexation-induced chemical shifts disappear in the ^1^H NMR spectrum upon addition of KI indicating a reversible dis-assembly. In addition, these calix[4]crown-4s have shown excellent Na^+^/K^+^ ion binding selectivity (K_a_ (Na^+^) = 1181 M^-1^ and K_a_ (K^+^) is negligible) which is attributed to the better cavity size matching of sodium ion than potassium ion.

Recently, Chawla *et al.* [76] have developed tetrathiacalix[4]arene-based receptors **7a-d** by incorporating arylazo, thiazoleazo, pyridylazo, and *β*-naphthylazo groups onto tetrathiacalix[4]arene which exhibit Cs^+^ and Rb^+^ ions selectivity over other alkali and alkaline earth metal ions. They observed bathochromic shifts in the UV-vis spectroscopy as well as significant color change in the visible region when the receptor interacted with these ions. It was believed that the cavity size of these azo-coupled tetrathiacalix[4]arenes played an important role in binding to the appropriate size of alkali metal ions. The study shows that these macrocyclic molecules can potentially be developed into useful ionic filters and sensor materials.


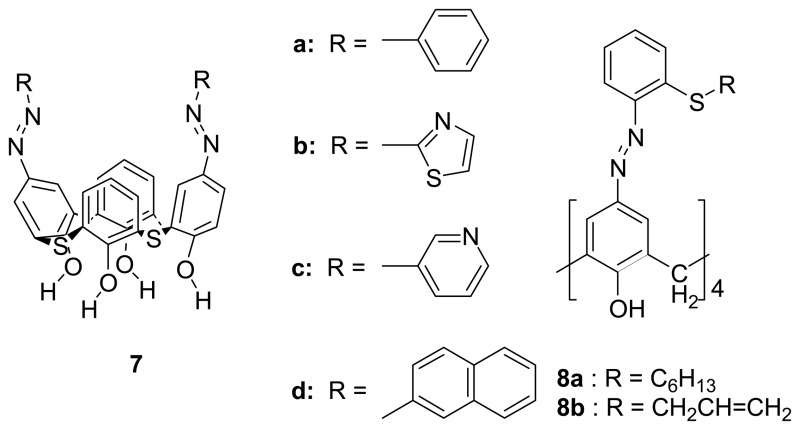


Chawla *et al.* [77] have also showed that **8a** and **8b** can detect a tiny amount (in ppm scale) of soft metal ions including Hg^2+^ and Pd^2+^ by a visual change. An immediate color change from yellow to red upon addition of Hg(OAc)_2_ and yellow to light purple upon addition of K_2_PdCl_4_ were observed. Substantial shifts in the *v_N_*_=_*_N_* and *v_S-C_* stretching frequency absorption bands in the IR spectra of complexed calixarene derivatives indicate that the coordination site includes the azo moiety as well as the sulphur atom of the thioalkyl group. They pointed out that these two chromogenic calixarenes containing multi-heteroatoms with the extended azobenzene fragments show a potential to develop into a molecular diagnostic tool using the UV-vis spectroscopy.

Chawla *et al.* also indicated the heterocyclic calix[4]arene **9a** with imidazole moieties on the upper rims is highly selective extractant for Ag^+^ ions. IR and ^1^H NMR studies indicated that silver ions interacted at the upper rim and the complexation process did not involve the hydrogen atoms attached to the nitrogens of the imidazole rings. A model study using monomer derivatives **9b** further suggested that the size and nature of this macrocyclic structure played an important role in the recognition process.


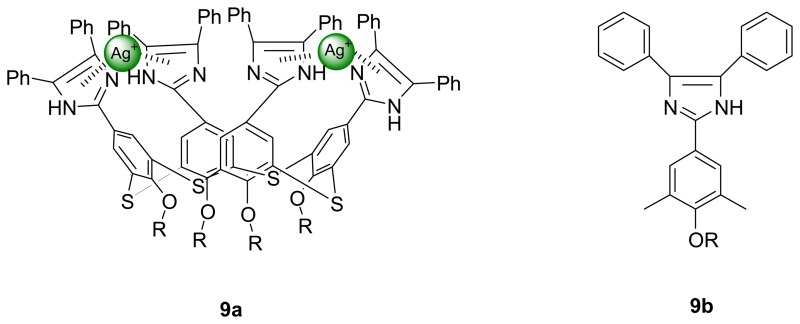


Kim *et al.* [78] have reported a dual sensing probe bis-diazophenyl substituted calix[4]crown-6 **10** which shows a high Pd^2+^ ion selectivity over other metal ions in acetonitrile. The spectral change in the absorption spectrum upon binding arises from the blue shift of azo groups on the upper rim which is due to the positively polarized phenolic oxygen atoms of the crown loop. The Pd^2+^ ion sensing was also apparent from the selective color change of this sensor from pale green to colorless. In addition, there is a fluorescence enhancement of the pyrenyl moieties via a suppressed fluorescence resonance energy transfer (FRET) upon complexation.


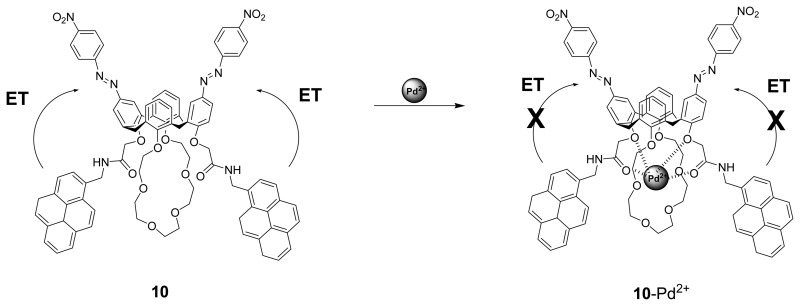


By elaborating on his previous work, Kim *et al.* [79] reported chromogenic cone-conformed tetrakis-diazophenylcalix[4]arene **11**, which showed a color change from yellow to orange, upon addition of transition metal ions such as Pd^2+^, Cr^3+^, Fe^2+^, Co^2+^ and Ni^2+^ ions. On the other hand, there is no significant spectral change upon the interaction with alkali and alkaline earth metal ions. The binding stoichiometry of 1:1 between **11** and Cr^3+^ was determined by the Job's experiment of continuous variation. The binding interaction is believed to be due to the release of protons from the azophenols to the quinone-hydrazone tautomer upon metal ion complexation, followed by internal chelation of metal ion to nitrogen atoms and ortho-ester carbonyl groups.


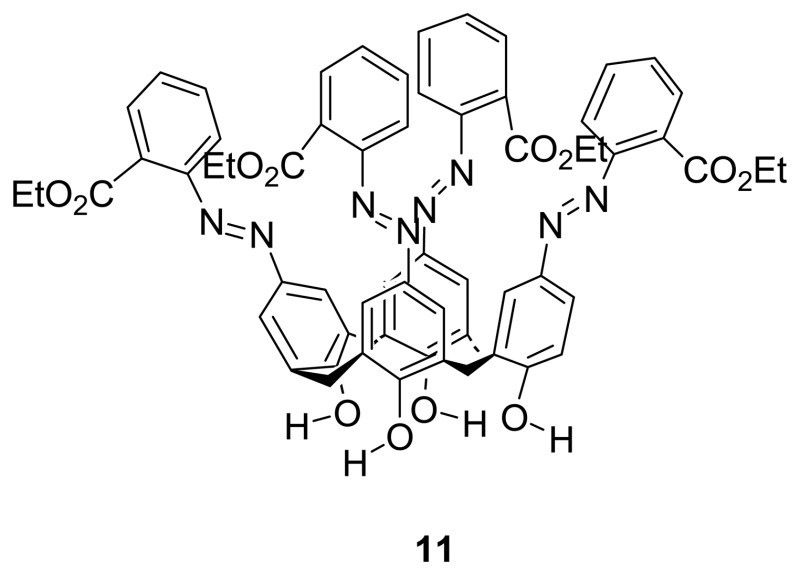


Kim *et al.* [80-82] have also designed and synthesized a series of extended homooxacalix[4]arene bearing tetraamides. The C-1,2-alternate conformer of tetrahomodioxacalix[4]arene *N*,*N*-dialkyl tetraamide **12a-b** exhibits selective binding towards Pd^2+^ ion over alkali, alkaline earth, transition metal and ammonium ions. From the X-ray structure of **12a**·Pd^2+^complex, it was found that Pd^2+^ ion was encapsulated by the carbonyl oxygen atoms of two adjacent amide groups and an oxygen atom of one of aryl-alkyl ethers. They also found that the C-1,3-alternate conformer **13** and the C-1,2-alternate conformer **12c** gave low extractability towards Pd^2+^ ion because of an intramolecular hydrogen bonding between N-H and oxygen atoms of the carbonyl or the phenyloxy groups. Their work could provide a guideline to optimize the conformation of homooxadioxacalix[4]arene derivatives for stronger metal ion binding.


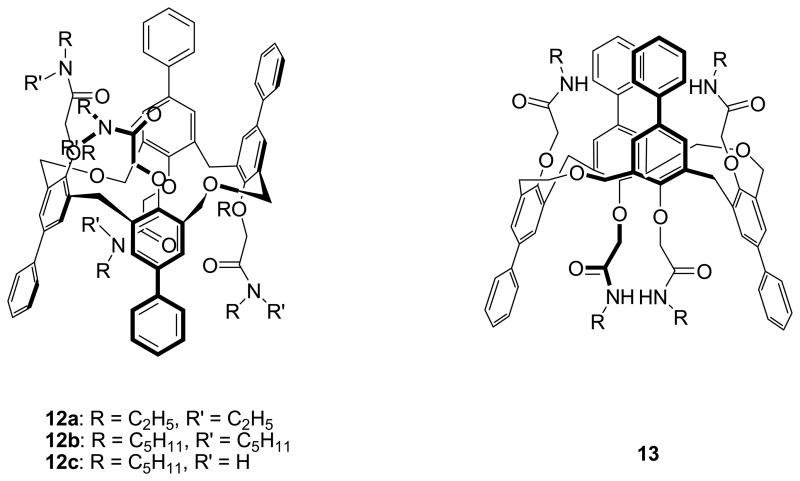


Alkaline earth metal ions such as Mg^2+^ and Ca^2+^ are the most important metal ions in biological systems. It is necessary to differentiate one from the other in order to achieve the clinical requirements. Over the past few years, many of calix[4]arene derivatives exhibit higher selectivity for alkali metal cations than alkaline earth metal cations and only a few calix[4]arene-based Ca^2+^ have been reported. Recently, attention has been drawn in using extended calix[4]arene derivatives to develop sensors for Ca^2+^ ion.

Xu *et al.* [83] have reported the synthesis of Schiff base-linked bis-anthracene substituted calix[4]arene **14**. This calix[4]arene receptor was found to show highly selectively interaction with Ca^2+^ ion in MeCN-methanol mixture over other alkali and alkaline-earth metal ions with which the most intense enhancement of fluorescence of about 260 times relative to the free sensor was induced upon Ca^2+^ binding. As this receptor undergoes photoinduced electron transfer (PET) from the lone-pair of the nitrogen atom of Schiff base linkage to the anthracene moiety, the fluorescence is quenched. However, the PET is inhibited and the fluorescence is restored when the lone-pair of nitrogen atom is involved in the coordination with a metal ion. The oxidation potential of the Schiff base functional group also increases substantially upon binding.


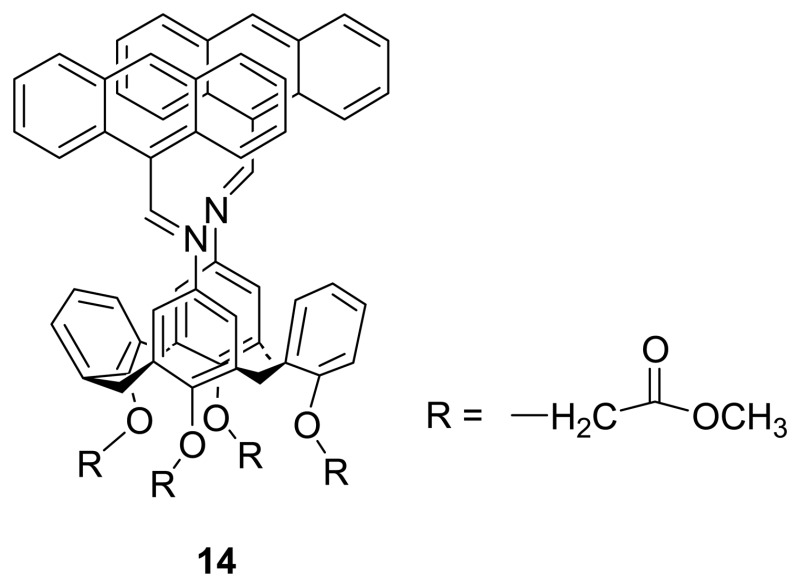


Subsequently, Kim *et al.* [84] also revealed bis(indoly)calix[4]crown-6, **15** in an oxidized form exhibiting selective recognition behaviors for both Ca^2+^ and F^-^ ions in CH_3_CN. In the presence of F^-^ion, the proton chemical shifts of this calixcrown-6 unit is shifted further by an addition of Ca^2+^ ion. The results of the titration experiments of free **15** and **15**·F^-^ with Ca^2+^ ion suggest that the calix[4]crown-6 binds stronger toward Ca^2+^ ion in the presence of F^-^ ion. Based on the UV-vis spectral change as well as the color change of **15,** they have proposed that F^-^ ion binds to the hydrogen atoms of the expanded oxidized bisindole units in its deprotonated state by hydrogen bonds. The binding stoichiometry of 1:2 between **15** and F^-^ was found by the Job's plot experiment. Interestingly, **15** also shows a selective color change upon addition of HSO_4_^-^ and H_2_PO4^-^ ions in H_2_O/CH_3_CN (1:1 v/v). Thus, sensor **15** has a potential to be used in three combinational NOR logic gates.


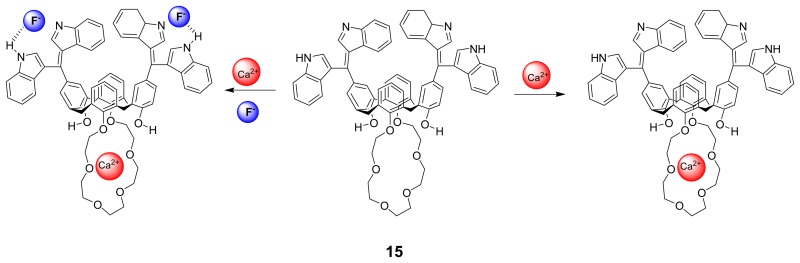


Shinkai *et al.* [85] have reported the synthesis and alkali metal ion binding behavior of bis-pyrene substituted calix[4]crown-4, **16**. Upon binding of a metal ion to the crown ether cavity, the two non-bridged distal phenol units become flattened in order to interact with the bound metal cation which would thus change the distance between two appended pyrene moieties at the upper rim. Such a change in distance would give rise to a large change in the monomer/excimer fluorescence intensity ratio. They have found that **16** bound with Na^+^ and Li^+^ but not with K^+^. In addition, they showed that the two distal pyrene moieties could interact with trinitrobenzene as there was an upfield shift (Δδ = 0.4 ppm) of aromatic proton resonance of trinitrobenzene in the ^1^H NMR spectrum upon its addition. On the other hand, the proton peak shift is slightly restored by adding the Na^+^ salt, implying the **16**·trinitrobenzene complex could be dissociated through metal-binding process.


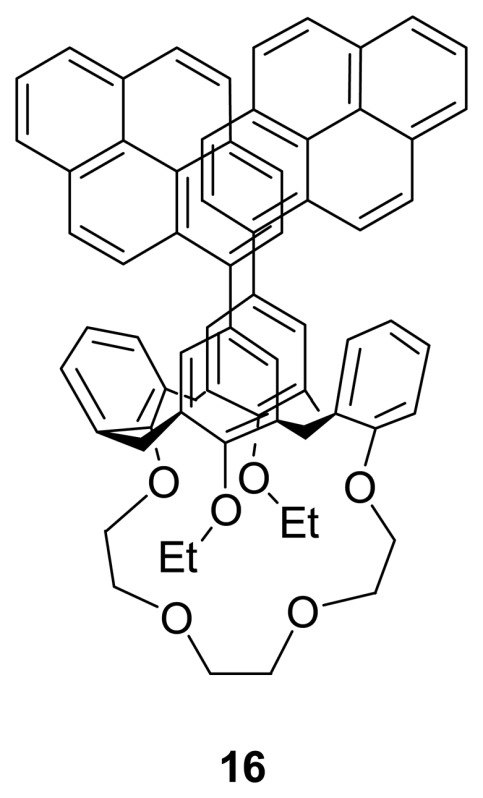


Takahashi *et al.* [86] have designed and synthesized novel calix[4]crown-4, **17** and calix[4]crown-4, **18** with the thienylene analogue of *p*-terphenoquinone as extended chromophore, which is known to exhibit strong absorption in the visible region and form a very stable radical anion and radical cation in the electrochemical reactions, for chromogenic and electrochemical sensing. Upon binding to Na^+^ or K^+^ ion, there is a dramatic conformational change of **17** and **18** from the 1,3-alternate conformer to the cone conformer which leads to a strong through-space exciton coupling of the two diametrically arranged chromophores, resulting in a significant color change. In addition, the crown-4-bridged **17** with a smaller ionophoric cavity exhibits a 100-fold higher selectivity for Na^+^ versus K^+^ than the crown-5-bridged **18**. Furthermore, **17** and **18** can show not only anodic shift in the reduction potential but also cathodic shift in the oxidation potential upon addition of Na^+^ or K^+^ ion indicating that their binding behavior can be monitored electrochemically.


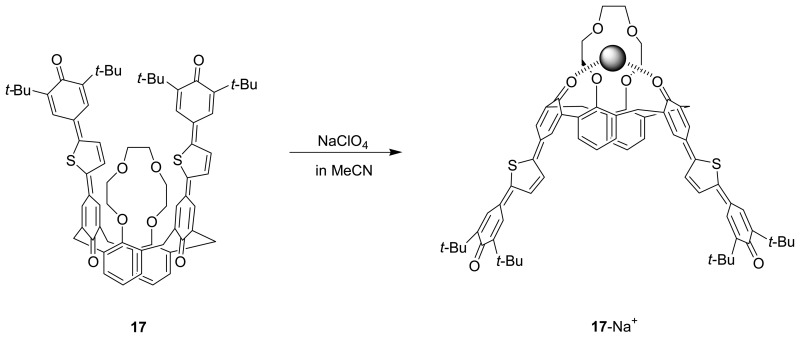
 
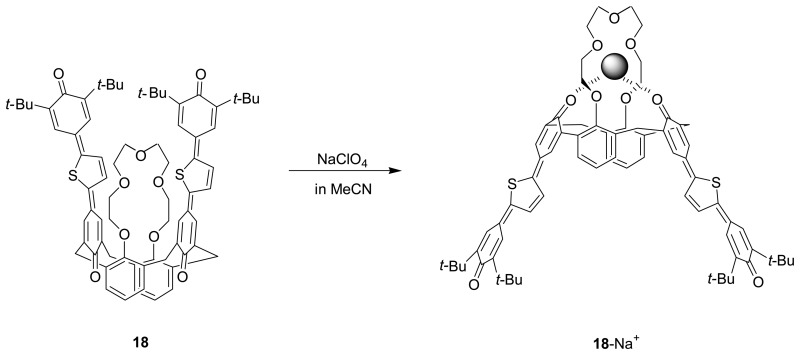


Rebek *et al.* [87-88] showed that expanded calix[4]arene tetraurea **19** in the cone conformation reversibly dimerized in apolar solvents such as CDCl_3_ or CD_2_Cl_2_ forming well-defined capsules, as revealed by ^1^H-NMR and ESI-MS spectroscopies. This dimer exhibit a large dimerization constant (K_a_ > 10^5^ M^-1^) and are kinetically stable in the ^1^H NMR time scale. The dimerization occurs via the formation of a junction of 16 C=O⋯N–H intermolecular hydrogen bonds (eight strong and eight weak) at the upper rim. The expanded calix[4]arene tetraurea capsule creates an egg-shaped cavity with estimated volume of ∼ 400 Å^3^ with which it can accommodate at least two benzene- or chloroform-sized guest molecules according to the molecular modeling. This expanded capsule also forms complexes with Na^+^ cation in the apolar solvents in which the oxygen atoms of four carbonyls and four phenolic ethers at the lower rim provide enough space for tightly coordination of Na^+^ cation. The solvent exchange rate in this cavity was found to be very fast as this expanded calix[4]arene tetraurea possesses a larger room which could allow solvent molecules to pass in and out of the capsule rapidly. Although the neutral guest encapsulation is not successful, encapsulation of the positively charged molecules **i-v** by the dimeric capsules (**19**·**19**) is strong which is attributed to the strong ion-dipole interactions.


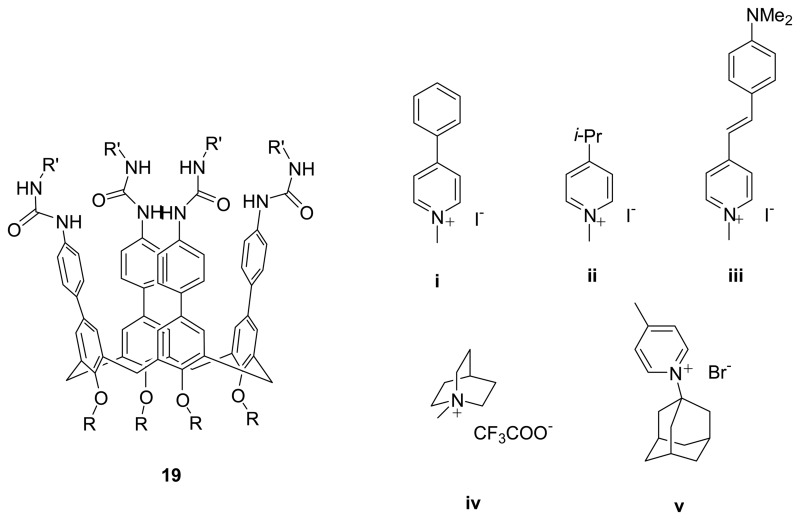


### 2.2. Extended Calix[4] arene-Based Receptors for Anions

Many biological processes in living organisms such as chemical transportation and transformation are strongly dependent of the presence or use of anions i.e. ATP and DNA. To date, anions are also significant for many industrial processes and often found as harmful pollutants such as phosphate and nitrate, which lead to entrophication. As a result, it is of utmost importance to develop useful anion sensors for different practical applications such as for cells, serum, blood, soil, and freshwater. However, it is still a great challenge to the scientific community to design and synthesize an artificial calix[4]arene-based receptors that show high binding affinity and selectivity to a targeted anion because anions have diverse topology or geometries. In addition, selective anionic receptors are relatively difficult to design and synthesize. In recent years, anion receptors based on an extended π-conjugated calix[4]arene skeleton have been developed which rely on a wide range of non-covalent interactions such as hydrogen-bonding, ion pairing or metal coordination for recognition.


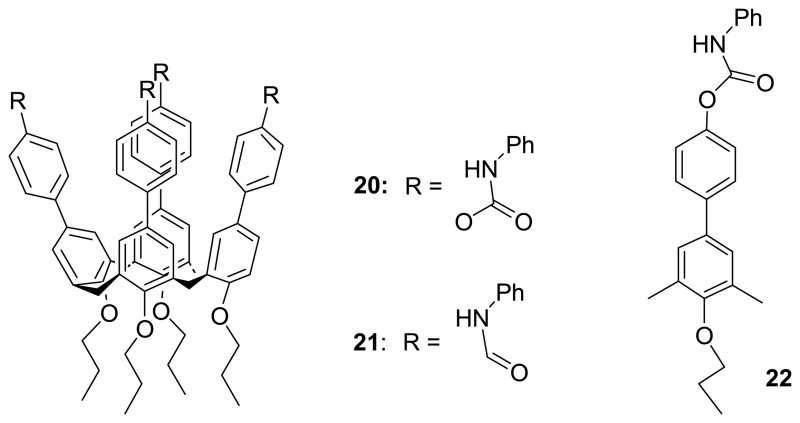


**Figure 8.**
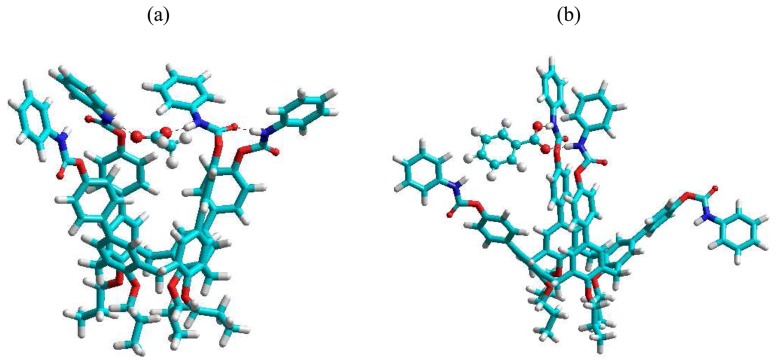
The optimized structures of (a) 20·CH_3_COO^-^ and (b) 20·Ph-COO^-^ complexes obtained by B3LYP 6-31G DFT calculations.

Recently, Wong *et al.* [89] reported the synthesis and sensing properties of **20**, **21**, and **22** with various anions (Bu_4_N^+^X^-^) including F^-^, CH_3_COO^-^, H_2_PO_4_^-^, and Ph^-^COO^-^ in which they were investigated by fluorescence titrations, ESI-MS measurements, and ^1^H NMR spectroscopies. They have demonstrated that carbamoylphenyl-substituted calix[4]arene **20** shows greatly enhanced binding affinity and selectively toward these anions as compared to the monomeric receptors **22**, particularly for carboxylates. Amidophenyl-substituted calix[4]arene **21** also exhibits superior binding selectivity for acetate ion over fluoride ion even though it has a relatively weaker binding affinity compared to that of **20**. In addition, both of these aryl-substituted calix[4]arene fluorescent receptors show much higher selectivity towards acetate over benzoate anion (for **20**: K_acetate/benzoate_ ∼ 12 and for **21**: K_acetate/benzoate_ > 10^4^). It has been pointed out that the binding and selectivity enhancement of these arylcalix[4]arene-based sensors are attributed to the cooperative binding of the multiple ligating functional units such as carbamoyl functionalities in **20** as revealed from ab inito B3LYP 6-31G DFT quantum mechanical calculations (Figure 8). The cooperative (or bidentate) binding mode in which the hydrogen bonds formed between carboxylate and the two opposite NH groups of carbamoyl moieties flanked from the extended calix[4]arene skeleton was consistently found in the optimized structures for both **20**·carboxylate complexes.

### 2.3. Extended Calix[4]arene-Based Receptors for Neutral Molecules

The first class of synthetic receptors for neutral molecules both in apolar solution and solid state was derived from calixarene[4]resorcinols. Recently, it has shown that the cone and 1,3-alternate conformers of calix[4]arene possess an intramolecular cavity which can host neutral guest molecules of complementary size. It has also established that the efficiency of the recognition process is strongly influenced by the rigidity of the host and the natures of the guests. Thus, calix[4]arene incorporated with extended or expanded π-conjugated framework has been developed to provide a much rigid skeleton to promote the binding and encapsulation of guests.

Fukazawa *et al.* [90-91] have reported the design and synthesis of extended monodeoxycalix[4]arene-based receptors **23** and **24** bearing two ethynylbenzoic acid moieties as the guest binding sites at the upper rim. The receptor **23** showed strong binding towards cyclic and acyclic ureas in CDCl_3_ by the interactions of hydrogen bonds with a 1:1 binding stoichiometry as determined the ^1^H NMR titration experiment and the Job's plot analysis. [90] They have also showed that the conformation of monodeoxycalix[4]crown-5, **24** can be modified upon binding of its crown ether with an appropriate alkali metal cation i.e. Na^+^ ion. Such conformation change can lead to an enhanced binding of this receptor with the neutral cyclic and acyclic monoalkylated urea guests. [91] The enhancement is attributed to the disruption of intramolecular hydrogen bonds between the two appended benzoic acids and more favorable attractive hydrogen bonding interactions between the receptor and guest.

Rathore *et al.* [92] have designed and synthesized a 1,3-alternate calix[4]arene ether derivative **25** as an electron-deficient receptor upon a chemical or an electrochemical oxidation for sensing of an electron-rich guest such as nitric oxide (NO) which is vital to biological system. The 1,3-alternate calix[4]arene **25** is easily electrochemically oxidized exhibiting a low, reversible first oxidation potential in the CV measurement corresponding to the formation of a stable electron-poor cation radical and also readily chemically oxidized by MA^+^ SbCl_6_^-^ (MA^+^ = 9,10-dimethoxyocahydro-1,4:5,8-dimethanoanthrancene cation radical). Upon exposure of **25**^+^ to gaseous NO, intense color change from bright green to dark blue is observed indicating the interaction of the two species. The **25** cation radical binds to a molecule of NO with a high affinity (K_NO_ > 10^8^ M^-1^) as estimated by Venus fly trap according to the competition method. The X-ray crystallography analysis confirms that the NO molecule is deeply encapsulated inside 1,3-alternate calix[4]arene cavity.


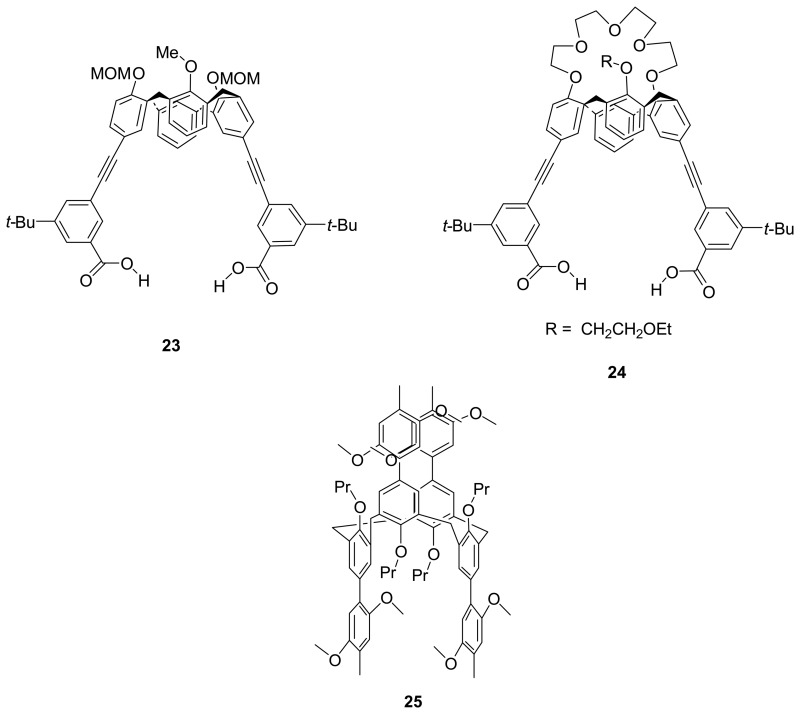


Mendoza *et al.* [93] synthesized an extended calix[4]arene **26** having the wide, coplanar 1*H*-phenanthro[9,10-d]imidazol-2-yl aromatic surface situated at the upper rim. They demonstrated that an expanded cavity of **26** could be created by hydrogen-bonded bridges or ion pairing. Upon addition of the hydrogen bond donors such as water, DMSO or hydrogen bond acceptors such as oxoanions (e.g. TFA), the slow tautomeric equilibrium, which was confirmed by splitting of the protons on the imidazole rings, as well as the well-resolved structure in solution indicated the presence of the four hydrogen-bonded or ion-paired bridges preventing the rotation of the phenanthroimidazole groups on the upper rim of the calix[4]arene platform. In the presence of monomeric *N*-methyl derivative **27**, there is a significant upfield shift of the ^1^H-NMR signal for the aromatic CH protons of **26** in CDCl_3_/TFA. In addition, the *N*-methyl signal of the monomer exhibits a dramatic upfield shift at 233 K, consistently, indicating that **25** is likely inside the expanded cavity.


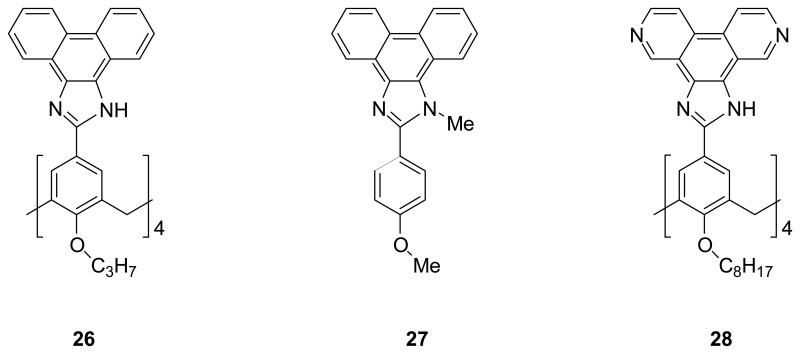


Subsequently, they have designed and synthesized the analogous **28** by replacing the phenanthrene moiety with 3,8-phenanthroline unit in order to introduce the heteroatoms at the corners of a square with the 90° angles for metal ion coordination. The reaction of **28** and [Re(CO)_5_Br] gives rise to a metallocavitand **28**·Re_4_ complex [94] which has been characterized by ESI-MS, IR and NMR spectroscopies. This metallocavitand exhibits a deep, square pyramidal cavity which can fit for molecules of complementary size and shape. The binding behavior of the metallocavitand with unsubstituted calix[4]arenes and cavitands has also been investigated. It has been found that the driving force to enclose the guest inside the deep metallocavitand originates from the face-to-face stacking interactions between the electron-rich guest and the electron-deficient metallohost which was in agreement with the results of the molecular modeling and the NMR studies.


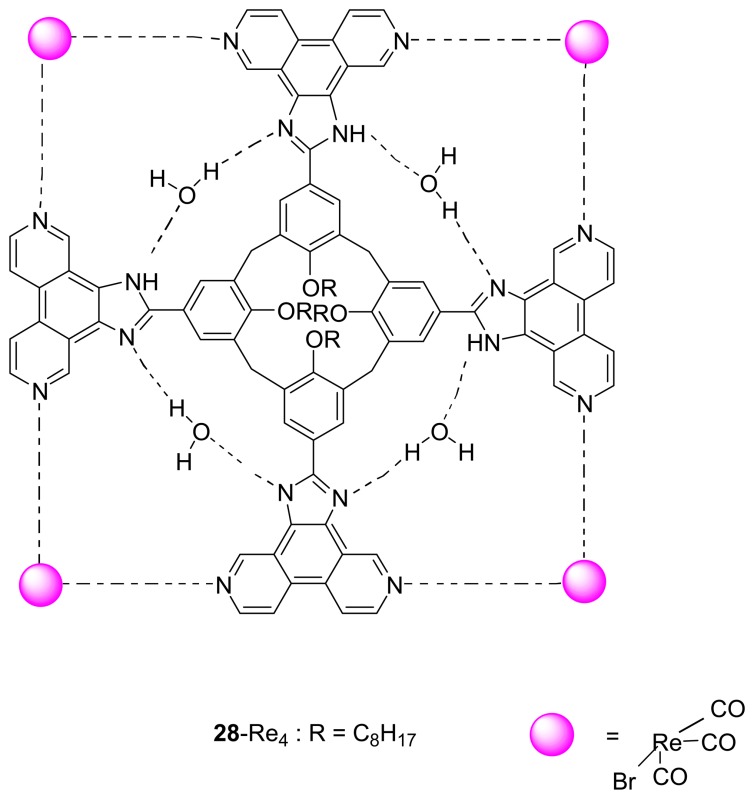


## 3. Conclusions

In view of the recent work, design of extended calix[4]arenes with multiple π-conjugated fluorophoric or chromophoric systems provides a useful strategy to enhance binding affinity, selectivity and sensitivity for recognition and sensing of a targeted ion or molecule. This review has shown that the rigid and pre-organized π-conjugated calix[4]arenes with multiple ligating groups and an expanded cavity can promote selectively cooperative binding for targeted guests and facilitate the enscapsulation of bigger guest molecules. For practical applications, progress still needs to be made to develop water-soluble expanded calixarenes for various sensing purposes. In fact, partially water-soluble expanded calixarenes could be enough for some analytical and environmental tests. Nevertheless, expanded calixarenes still remains a largely unexplored approach to design effective and useful artificial receptors particularly for anions and biomolecules as well as supramolecular systems.
